# The evaluation of urinary calprotectin levels for prediction of acute cisplatin-induced nephrotoxicity

**DOI:** 10.1097/MD.0000000000029814

**Published:** 2022-06-30

**Authors:** Gülay Koçak, Gamze Bilik, Aylia Yeşilova, Firat Oyman, Murat Can, Şener Cihan

**Affiliations:** a Department of Nephrology, University of Health Sciences, Prof. Dr. Cemil Taşcioğlu City Hospital, İstanbul, Turkey; b Department of Internal Medicine, University of Health Sciences, Prof. Dr. Cemil Taşcioğlu City Hospital, İstanbul, Turkey; c Department of Biochemistry, Bülent Ecevit University, Faculty of Medicine, Zonguldak, Turkey; d Department of Medical Oncology, University of Health Sciences, Prof. Dr. Cemil Taşcioğlu City Hospital, İstanbul, Turkey.

**Keywords:** cisplatin, non-small cell lung cancer, urinary calprotectin

## Abstract

Calprotectin is a protein molecule that is released from inflammatory cells. Measurement of calprotectin in various body fluids has recently gained significant importance for differentiating inflammatory and noninflammatory events. The subject has aroused interest in the field of nephrology and some renal pathologies in which urinary calprotectin levels have been studied. In this study, the measurement of urinary calprotectin level and its use for determining acute cisplatin nephrotoxicity in a group of patients with non-small cell lung cancer who received cisplatin-based oncological treatments have been investigated.

The study included 41 patients who received cisplatin-based treatments for non-small cell lung cancer between January 2019 and January 2020. The patients were excluded from this study who were with estimated glomerular filtration rate (eGFR) <60 mL/min/1.73 m^2^, serum creatinine (sCr) >1.5 mg/dL, a history of urinary tract infection, and nephrotoxic drug use in the past month. Baseline and 48-hour sCr values and baseline, 6-hour, 12-hour, 24-hour, and 48-hour urinary calprotectin levels of all patients were measured.

Four of the 41 patients who received cisplatin treatment were excluded because their 48-hour sCr values could not be accessed. The control group included 29 patients. While there was no difference between the cisplatin group and the control group in terms of baseline sCr and eGFR values, the cisplatin group had significantly higher urinary calprotectin values. Of the 37 patients treated with cisplatin, 7 (18.9%) developed cisplatin-induced nephrotoxicity. The comparison of groups with (group 1) and without cisplatin nephrotoxicity (group 2) showed comparable mean age and male sex ratio. Baseline sCr and eGFR values were similar in both groups. The cisplatin-induced nephrotoxicity group had significantly higher 48-hour sCr and significantly lower 48-hour eGFR values. Baseline, 12-hour, 24-hour, and 48-hour urinary calprotectin levels were similar in groups with and without cisplatin nephrotoxicity.

Recent studies have demonstrated that urinary calprotectin level measurement can be used to distinguish intrinsic acute kidney disease from prerenal kidney disease. However, the comparison of groups with and without cisplatin nephrotoxicity in our study showed no difference in urinary calprotectin levels. However, there is a need for large-scale studies using combined urinary biomarkers.

## 1. Introduction

Cisplatin, a platinum-based drug, is widely used in the treatment of many cancers. However, the nephrotoxicity induced by cisplatin is a common and unwanted adverse effect, which has significantly restricted its clinical use.^[1]^ Current clinical measures, such as serum creatinine (sCr) and estimated glomerular filtration rate, are inadequate for early detection of acute kidney injury (AKI), especially when there is only a slight to moderate damage. Therefore, the identification of sensitive urinary biomarker associated with the ischemic or nephrotoxic AKI such as cisplatin-induced nephrotoxicity is a subject of interest in nephrology practice.

Calprotectin is a protein-structured molecule, which has a physiological function in the immune system.^[2]^ The fecal calprotectin concentration has long been used as a reliable parameter in differential diagnosis of inflammatory bowel disease. Recently, it has been defined that urinary calprotectin levels can be used to make a distinction between prerenal and intrinsic AKI.^[3]^ In this study, we aimed to evaluate whether the urinary calprotectin level may predict acute cisplatin-induced nephrotoxicity in patients with non-small cell lung cancer.

## 2. Material and method

### 2.1. Patients and study setting

We performed a prospective study between January 2019 and January 2020. All patients who have history of non-small cell lung cancer (NSCLC) were evaluated according to the inclusion criteria of our study. The patients with NSCLC who received cisplatin-based therapy, who were >18 years of age, and who gave informed consent for data collection were included in the study. Patients with estimated glomerular filtration rate (eGFR) <60 mL/min/1.73 m^2^ or blood creatinine level >1.5 mgr/dL, received medication associated with nephrotoxicity such as aminoglycosides and amphotericin B, or history of urinary tract infection within the last month period were excluded.

All patients received a single dose of cisplatin. A single dose of cisplatin at a dose of 75 mg/m^2^ was administrated intravenously by 1000 mL isotonic saline over 1 hour. Two grams of magnesium sulfate and 20 mEq of potassium chloride were infused at 1 hour within 1000 mL isotonic saline after cisplatin administration for preventing the nephrotoxicity. As the amount of intravenous fluid due to treatment of cisplatin may alter urinary calprotectin levels, all patients received an average of 2.5 L of intravenous saline therapy (between minimum 2450 mL daily and maximum 3200 mL daily).

The study was conducted with the approval of the Clinical Research Ethics Committee of our local hospital, dated October 9, 2018, and numbered 1008.

### 2.2. Data collection and assessment

Renal function was evaluated by the sCr and the eGFR value calculated using the Chronic Kidney Disease Epidemiology Collaboration formula. The serum samples were collected at baseline and at 48 hours of cisplatin administration. The presence of acute nephrotoxicity associated with cisplatin administration was defined according to the Acute Kidney Injury Network criteria.^[4]^ Acute nephrotoxicity was defined as a >0.3 mg/dL rise in sCr or an increase of 1.5- to 2-fold from baseline at 48 hours of cisplatin administration.

The urinary samples were collected in sterile bottles at baseline and at 6,12, 24, 48 hours of cisplatin administration. Urinary samples were preserved in refrigerator (−80°C) until the time of assay. Urinary calprotectin levels were measured by a sandwich enzyme-linked immunosorbent assay technique using reagents provided by universal service control number Business Co Ltd, Wuhan, China. The detection range of urinary calprotectin was from 31.2 to 2000 pg/mL. The sensitivity was <13.3 pg/mL.

### 2.3. Statistical analysis

Data were presented as the mean ± standard deviation. A statistical analysis was performed using SPSS version 21.0 (SPSS Inc., Chicago, IL). Basic descriptive statistics were measured, including the means, standard deviations, ranges, and percentages. The normality of the distribution was examined by the Kolmogorov–Smirnov test. Mean values between 2 independent groups were compared by the Mann–Whitney *U* test for continuous variables and by the χ^2^ test for categorical parameters; comparisons between >2 subgroups were performed by ANOVA and Kruskal–Wallis H tests. Differences were considered to be statistically significant if the 2-tailed *P* value was <.05.

## 3. Results

The study included 41 patients who received cisplatin-based treatments for NSCLC in the medical oncology department of our hospital between January 2019 and January 2020. Four of these patients were excluded from the study because of inaccessible 48-hour sCr values or inadequate urine samples obtained at 6-hour intervals. The control group included 29 patients. The mean age of the cisplatin group was 63.27 ± 7.82 years, while the mean age of the control group was 40.73 ± 8.0 years, with a statistically significant difference (*P* = .001). The male sex ratio of the cisplatin group was higher, with a significant difference (*P* = .001). While there was no difference between the cisplatin group and the control group in terms of baseline sCr and eGFR values, the cisplatin group had significantly higher urinary calprotectin values (*P* = .001). Baseline demographic characteristics and laboratory data of the study and control groups are presented in Table 1.

**Table 1:** Baseline demographic and laboratory characteristics of all patients.

Characteristics	Cisplatin group (n = 37)	Control (n = 29)	*P* value
Age (yr)	63.27 + 7.82	40.73 + 8.0	.001*
Male	7 F/30 M	16 F/13 M	.001*
(n, %)	18.9%/81.1%	55.2%/44.8%	
Baseline sCr (mg/dL)	0.73 + 0.14	0.69 + 0.16	.4
Baseline eGFR (mL/min/1.73 m^2^ )	96.35 + 11.87	112.23 + 12.01	.001*
Baseline urinary calprotectin (pg/mL)	435.77 + 209.43	135.48 + 58.81	.001*

eGFR = estimated glomerular filtration rate, sCr = serum creatinine.

* *P* < .05.

Of the 37 patients treated with cisplatin, 7 (18.9%) developed cisplatin-induced nephrotoxicity. The comparison of groups with (group 1) and without cisplatin-induced nephrotoxicity (group 2) showed no difference according to age. Baseline sCr and eGFR values were similar in both groups. The cisplatin-induced nephrotoxicity group had significantly higher 48-hour sCr and significantly lower 48-hour eGFR values (*P* = .001, in comparison of groups for both parameters). Baseline and 48-hour urinary calprotectin levels were similar in groups with and without cisplatin nephrotoxicity (Table 2).

**Table 2:** Demographic and laboratory characteristics of the study population.

Characteristics	Group 1: cisplatin-administrated patients with nephrotoxicity (n = 7)	Group 2: cisplatin-administrated patients without nephrotoxicity (n = 30)	*P* value
Age (yr)	65.42 ± 8.5	62.76 ± 7.70	.4
Male (n, %)	4 M 57.1% /3 F 42.9%	26 M % 86.7 / 4 F % 13.3	.01*
Baseline sCr (mg/dL)	0.78 ± 0.14	0.72 ± 0.15	.3
Baseline eGFR (mL/min/1.73 m^2^ )	87.86 ± 14.9	98.56 ± 10.12	003
sCr at 48. H (mg/dL)	1.11 ± 0.12	0.74 ± 0.16	.001*
eGFR at 48. H (ml/min/1.73 m^2^ )	62.14 ± 12.36	97.34 ± 12.30	.001*
Urinary calprotectin baseline (pg/mL)	419.00 ± 242.12	439.96 ± 205.21	.8
Urinary calprotectin at 6 hs (pg/mL)	364.00 ± 213.04	405.64 ± 264 .21	.6
Urinary calprotectin at 12 h (pg/mL)	302.57 ± 214.70	405.57 ± 268.021	.3
Urinary calprotectin at 24 h (pg/mL)	381.29 ± 227.25	419.74 ± 236.62	.7
Urinary calprotectin at 48 h (pg/mL)	428.71 ± 215.94	426.27 ± 258.55	.9

eGFR = estimated glomerular filtration rate, F = female, M = male, sCr = serum creatinine.

* *P* < .05.

**Figure 1.**
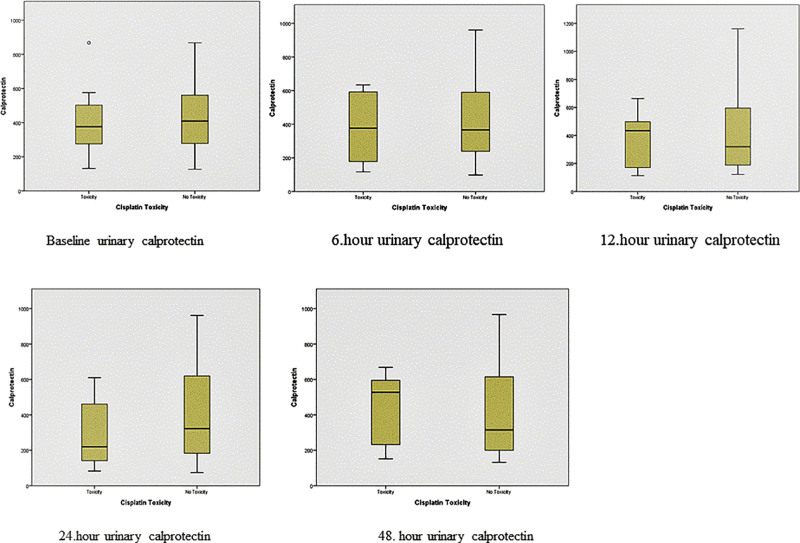
The evaluation of groups in terms of urinary calprotectin values from baseline to 48 h.

The evaluation of groups with (group 1) and without cisplatin nephrotoxicity (group 2) in terms of urinary calprotectin values calculated at baseline, 6, 12, 24, and 48 hours showed no difference between the groups (Fig. 1).

## 4. Discussion

In our study, we found that urinary calprotectin levels in patients with NSCLC were significantly higher than healthy control. However, the baseline and intermittent measurements of urinary calprotectin levels were not significantly different between patients with and without acute cisplatin-induced nephrotoxicity.

Calprotectin, a heterodimeric protein complex, has calcium and zinc ion-binding property. These ions are essential for bacterial growth and their chelations by calprotectin may contribute to the antimicrobial effect. Since the increase of sCr has been observed in the late stages of AKI, urinary markers that will determine the damage in the early stage have attracted particular attention from the nephrologists for a long time. Although calprotectin is mainly located in the neutrophils, it has also been shown in monocytes and macrophages.^[2,5]^ It is mainly released by neutrophils during inflammation. Therefore, the detection of calprotectin levels in serum and various body fluids to distinguish inflammatory disease from noninflammatory disease has been used for a while.^[5]^

In addition to evaluating sCr and urinary output, using various biomarkers may help physician in the early detection, differential diagnosis, and prognostic assessment of AKI.^[6]^ First observation regarding the involvement of calprotectin in renal pathology was performed by Fujiu^[7]^ in a model of kidney injury by unilateral ureteral obstruction. It has been shown that calprotectin promotes the differentiation of monocytes to macrophages and causes epithelial injury and inflammation.^[8]^ Another study performed by Dessing et al^[9]^ showed that calprotectin is also induced in response to ischemia–reperfusion injury in mice. In the later studies, the diagnostic accuracy of urinary calprotectin levels in distinguishing prerenal from intrinsic AKI have been investigated.^[10–12]^ It has been observed that the increase in the urine calprotectin levels is a valuable parameter for discrimination of prerenal and intrinsic AKI in all of these studies.

Cisplatin used in the treatment of many solid-organ cancers has dose-limiting side effects such as nephrotoxicity. Because the increase in sCr value may be observed in the late stages of AKI, the urinary biomarkers such as neutrophil gelatinase-associated lipocalin and cystatin C have been studied for the early detection of cisplatin-induced nephrotoxicity.^[13]^ During the review of the literature, it was found that there were limited data on whether the evaluation of urinary calprotectin might be useful for the early detection of acute cisplatin nephrotoxicity. Kim et al^[14]^ showed that the urinary calprotectin, also called S100A8/A9, was increased in rat models with cisplatin-induced AKI. The serum and urinary calprotectin levels were simultaneously measured in 61 hospitalized patients with diagnosed AKI in another part of this experimental study. There was also an increase in the urinary calprotectin in hospitalized patients with intrinsic AKI.

In our study, the baseline urinary calprotectin levels of cisplatin received patients were significantly elevated than control, consistent with the current literature. Elevations of serum and urinary calprotectin levels were observed in some of the cancers such as breast, cervical, and squamous cell in the literature.^[15,16]^ Calprotectin has an important role in the metastasis and migration process of cancer cells by upregulation of vascular endothelial growth factor.^[17]^

We performed a prospective study to determine whether the early diagnosis of AKI using urinary calprotectin in NSCLC patients administered cisplatin. In our study, there was no significant difference in terms of the urinary calprotectin levels in patients with and without cisplatin-induced nephrotoxicity. Reviewing the literature, there was only 1 similar study found that was performed with an experimental design.^[13]^ On the other hand, urinary calprotectin levels have been recently evaluated in 490 patients undergoing coroner angiography and no significant difference was found for urinary calprotectin levels in patients who developed contrast-induced nephrotoxicity.^[17]^

In our study, urinary calprotectin levels were not increased in the cisplatin-induced nephrotoxicity group. It is known that cisplatin is especially toxic in proximal tubule of kidney. However, urinary calprotectin is mostly released from the distal part of the kidney, mainly collecting duct, and from immune cells infiltrating this region immediately after AKI.^[6]^ Another explanation may be that the patients in our study have a slight degree of AKI and this can be considered as the most important limitation of this study. Another limitation of this study is the small number of patients included. However, due to its prospective design and being performed in a specific patient group, we think that it enabled us to achieve important results.

In conclusion, it was shown that the measuring of urinary calprotectin levels did not provide early diagnosis of cisplatin nephrotoxicity in our study. However, further studies that also include other biomarker combinations in larger and heterogeneous patient populations are needed.

## Author contribution

Gülay Koçak: project development, manuscript writing, Gamze Bilik and Firat Oyman: data collection, Aylia Yeşilova: manuscript editing and data collection, Murat Can: analysis of urinary samples, Şener Cihan: manuscript editing and data analysis.

## References

[1] Barton CD Pizer B Jones C. Identifying cisplatin-induced kidney damage in paediatric oncology patients. Pediatr Nephrol. 2018;33:1467–74. 28821959 10.1007/s00467-017-3765-6 PMC6061670

[2] Stríz I Trebichavský I. Calprotectin—a pleiotropic molecule in acute and chronic inflammation. Physiol Res. 2004;53:245–53. 15209531

[3] Heller F Frischmann S Grünbaum M. Urinary calprotectin and the distinction between prerenal and intrinsic acute kidney injury. Clin J Am Soc Nephrol. 2011;6:2347–55. 21885792 10.2215/CJN.02490311 PMC3359561

[4] Mehta RL Kellum JA Shah SV. Acute Kidney Injury Network: report of an initiative to improve outcomes in acute kidney injury. Crit Care. 2007;11:R31. 17331245 10.1186/cc5713 PMC2206446

[5] Jeong SJ. The role of fecal calprotectin in pediatric disease. Korean J Pediatr. 2019;62:287–91. 30999729 10.3345/kjp.2019.00059 PMC6702112

[6] Schrezenmeier EV Barasch J Budde K. Biomarkers in acute kidney injury—pathophysiological basis and clinical performance. Acta Physiol. 2017;219:556–74. 10.1111/apha.12764 PMC5575831 27474473

[7] Fujiu K Manabe I Nagai R J Clin Invest. 2011;121:3425–41. 21821915 10.1172/JCI57582 PMC3163964

[8] Manabe I Nagai R. Renal collecting duct epithelial cells regulate inflammation in tubulointerstitial damage in mice. J Clin Invest. 2011;121:3425–41 21821915 10.1172/JCI57582 PMC3163964

[9] Dessing MC Tammaro A Pulskens WP. The calcium-binding protein complex S100A8/A9 has a crucial role in controlling macrophage-mediated renal repair following ischemia/reperfusion. Kidney Int. 2015;87:85–94. 24940802 10.1038/ki.2014.216

[10] Heller F Frischmann S Grünbaum M. Urinary calprotectin and the distinction between prerenal and intrinsic acute kidney injury. Clin J Am Soc Nephrol. 2011;6:2347–55. 21885792 10.2215/CJN.02490311 PMC3359561

[11] Seibert FS Pagonas N Arndt R. Calprotectin and neutrophil gelatinase-associated lipocalin in the differentiation of pre-renal and intrinsic acute kidney injury. Acta Physiol. 2013;207:700–8. 10.1111/apha.12064 23336369

[12] Chang CH Yang CH Yang HY. Urinary biomarkers improve the diagnosis of intrinsic acute kidney injury in coronary care units. Medicine (Baltimore). 2015;94:e1703. 26448023 10.1097/MD.0000000000001703 PMC4616771

[13] Tonomura Y Tsuchiya N Torii M. Evaluation of the usefulness of urinary biomarkers for nephrotoxicity in rats. Toxicology. 2010;273:53–9. 20438795 10.1016/j.tox.2010.04.015

[14] Kim AJ Ro H Kim H. Klotho and S100A8/A9 as discriminative markers between pre-renal and intrinsic acute kidney injury. PLoS One. 2016;11:e0147255. 26799323 10.1371/journal.pone.0147255 PMC4723127

[15] Arai K Takano S Teratani T. S100A8 and S100A9 overexpression is associated with poor pathological parameters in invasive ductal carcinoma of the breast. Curr Cancer Drug Targets. 2008;8:243–52. 18537548 10.2174/156800908784533445

[16] Zha H Li X Sun H. S100A9 promotes the proliferation and migration of cervical cancer cells by inducing epithelial-mesenchymal transition and activating the Wnt/beta-catenin pathway. Int J Oncol. 2019;55:35–44. 31059008 10.3892/ijo.2019.4793 PMC6561615

[17] Pan S Hu Y Hu M. S100A8 facilitates cholangiocarcinoma metastasis via upregulation of VEGF through TLR4/NF-kappaB pathway activation. Int J Oncol. 2020;56:101–12. 31746424 10.3892/ijo.2019.4907 PMC6910197

[18] Seibert FS Heringhaus A Pagonas N. Biomarkers in the prediction of contrast media induced nephropathy—the BITCOIN study. PLoS One. 2020;15:e0234921. 32673348 10.1371/journal.pone.0234921 PMC7365403

